# Impact of Salinity on the Gastrointestinal Bacterial Community of *Theodoxus fluviatilis*

**DOI:** 10.3389/fmicb.2020.00683

**Published:** 2020-05-08

**Authors:** Carmen Kivistik, Jan Knobloch, Kairi Käiro, Helen Tammert, Veljo Kisand, Jan-Peter Hildebrandt, Daniel P. R. Herlemann

**Affiliations:** ^1^Centre for Limnology, Estonian University of Life Sciences, Tartu, Estonia; ^2^Zoological Institute and Museum, University of Greifswald, Greifswald, Germany; ^3^Institute of Technology, University of Tartu, Tartu, Estonia

**Keywords:** microbiome, aquatic snail, brackish water system, 16S rRNA, planctomycetes

## Abstract

Differences in salinity are boundaries that act as barriers for the dispersal of most aquatic organisms. This creates distinctive biota in freshwater and brackish water (mesohaline) environments. To test how saline boundaries influence the diversity and composition of host-associated microbiota, we analyzed the microbiome within the digestive tract of *Theodoxus fluviatilis*, an organism able to cross the freshwater and mesohaline boundary. Alpha-diversity measures of the microbiome in freshwater and brackish water were not significantly different. However, the composition of the bacterial community within freshwater *T. fluviatilis* differed significantly compared with mesohaline *T. fluviatilis* and typical bacteria could be determined for the freshwater and the mesohaline digestive tract microbiome. An artificial increase in salinity surrounding these freshwater snails resulted in a strong change in the bacterial community and typical marine bacteria became more pronounced in the digestive tract microbiome of freshwater *T. fluviatilis*. However, the composition of the digestive tract microbiome in freshwater snails did not converge to that found within mesohaline snails. Within mesohaline snails, no cardinal change was found after either an increase or decrease in salinity. In all samples, *Pseudomonas*, *Pirellula*, *Flavobacterium*, *Limnohabitans*, and *Acinetobacter* were among the most abundant bacteria. These bacterial genera were largely unaffected by changes in environmental conditions. As permanent residents in *T. fluviatilis*, they may support the digestion of the algal food in the digestive tract. Our results show that freshwater and mesohaline water host-associated microbiomes respond differently to changes in salinity. Therefore, the salinization of coastal freshwater environments due to a rise in sea level can influence the gut microbiome and its functions with currently unknown consequences for, e.g., nutritional physiology of the host.

## Introduction

Salinity is among the most important environmental factors that determine the composition of aquatic microbial communities (Crump et al., 2004; Herlemann et al., 2011). A global-scale meta-analysis of samples from different habitats suggested that salinity is the major determinant of bacterial communities (Lozupone and Knight, 2007). Salinity also has a strong influence on the diversity of macrozoobenthic organisms (Remane, 1934). The gastropod *Theodoxus fluviatilis* is a widely distributed snail typically found in rivers and lakes as well as in brackish water in the coastal regions of Europe (Bunje, 2005). *T. fluviatilis* has the ability to live in fresh- and brackish waters up to a salinity 28 and thus are found in the western Baltic Sea (Bondesen, 1940; Zettler, 2008). The origin of the snail has been assumed to be the brackish Black Sea (Butenko, 2001).

The main food source of *T. fluviatilis* are diatoms (65%) (Neumann, 1959; Skoog, 1978) but also detritus (30%) and green algae (5%) (Calow, 1973; Skoog, 1978). Green algae have been considered a poor food supply for the snail because *T. fluviatilis* appears to lack the ability to digest cellulose (Neumann, 1961). However, previous studies have shown that many carbohydrases such as cellulase and chitinase in the digestive tract are supplied by the bacterial digestive tract community (Strasdine and Whitaker, 1963; Pinheiro et al., 2015). The gut microbiome has long been recognized as one of the most important sites of microbe/host interactions. The host protects beneficial microbes in the digestive tract and supplies them with food while the colonizing microbes provide the host with nutrients and detoxify secondary compounds within the food (Bhat et al., 1998; Dillon and Dillon, 2004). A stable gut microbiome can also protect the host from invasion by pathogenic exogenous microbes (Rolfe, 1997; Dillon and Dillon, 2004; Dong et al., 2009; Nicolai et al., 2015). Host factors that select for microbes include the host diet, anatomical structure of the gut, and the physical and chemical conditions of the digestive tract (Savage, 1977). Chemical conditions within the gut are usually kept relatively constant by the host. However, with respect to external salinity *T. fluviatilis* is an osmoconformer (Symanowski and Hildebrandt, 2010). Its presence in freshwater environments and brackish environments therefore also results in freshwater and brackish conditions within its digestive tract. Because salinity has a strong influence on bacterial communities, a change in salinity in the digestive tract would likely influence the bacterial community composition. Little is known about the influence of changing salinity in a host-protected system such as the digestive tract. Depending on the salinity tolerance, strict freshwater bacteria may be extinguished while saline tolerant bacteria may survive and marine bacteria could immigrate. Salinity shifts therefore also favors habitat generalists with a broad salinity tolerance (Székely and Langenheder, 2014). However, because *T. fluviatilis* is also highly abundant at mesohaline conditions, the gut microbiota of the snail must be able to cope with a change in salinity. Characterizing the responses to changing salinities within host associated microorganisms will help us understand how changing environmental conditions can influence the host-protected microbiome and reveal the mechanisms of host–microbiome interactions.

The main aim of this study was to determine if the host is able to maintain the original bacterial community and its functions during a shift to different salinities. To address this, we used a full-factorial design where *T. fluviatilis* obtained from freshwater was exposed to high salinities and *T. fluviatilis* obtained from mesohaline water was exposed to freshwater and polyhaline salinities. The response of the bacterial community in respect to their ambient state was monitored by 16S rRNA gene analysis. Our hypotheses were (1) *T. fluviatilis* obtained from different salinities have significantly different bacterial community compositions; (2) a hyperosmotic and hypoosmotic shift results in a change of the bacterial community toward brackish and freshwater bacterial communities, respectively; and (3) a set of core microorganisms (“core microbiome”) remains after a shift in salinity.

## Materials and Methods

### Sampling of Snails and Salinity Manipulations

*Theodoxus fluviatilis* snails were collected between July–October 2018 from three freshwater locations in Germany (Tables 1, 2 and Supplementary Figure S1). Lake Schmaler Luzin, Northern part—S1 (53°19′33″N, 13°26′28″E), Lake Schmaler Luzin, South end—S2 (53°18′01″N, 13°25′55″E), and Lake Carwitz—S3 (53°18′21″N, 13°26′48″E) and from two brackish water sites in the Baltic Sea: Ludwigsburg, Greifswalder Bodden—S5, salinity 8, (54°07′06″N, 13°28′35″E) and from Hiddensee, Vitter Bodden—S6, salinity 10 (54°34′40″N, 13°06′48″E).

**TABLE 1:** Overview of the freshwater sampling sites location, legend, coordinates, ambient and manipulation conditions, manipulation time, and Oligochaeta found in the shell of *Theodoxus fluviatilis.*

**Location**	**Location legend**	**Coordinates**	**Snails collected**	**Ambient salinity**	**Manipulation**	**Manipulation time (h)**	***Oligochatea* found**
Lake Schmaler Luzin, northern part	S1	53°19′33″N, 13°26′28″E	136	Freshwater Salinity 0.5	No manipulation	216	>5
Lake Schmaler Luzin, south end	S2	53°19′33″N, 13°26′29″E	136	Freshwater Salinity 0.5	**Hyperosmotic treatment**		2–5
					Salinity 0.5 (acclimatization to artificial water)	24	
					Salinity 6	48	
					Salinity 12	48	
					Salinity 18	120	
Lake Carwitzer	S3	53°18′21″N, 13°26′48″E	115	Freshwater Salinity 0.5	**Hyperosmotic treatment**		>5
					Salinity 0.5 (acclimatization to artificial water)	24	
					Salinity 6	48	
					Salinity 12	48	
					Salinity 18	120	

**TABLE 2:** Overview of the brackish water sampling sites location, legend, coordinates, ambient and manipulation conditions, manipulation time, and Oligochaeta found in the shell of *Theodoxus fluviatilis.*

**Location**	**Location legend**	**Coordinates**	**Snails collected**	**Ambient salinity**	**Manipulation**	**Manipulation time (h)**	***Oligochatea* found**
Ludwigsburg, Greifswalder Bodden	S5	54°6′59″N, 13°28′42″E	117	Mesohaline Salinity 8	**Hyperosmotic treatment**		0
					Salinity 8 (acclimatization to artificial water)	24	
					Salinity 16	48	
					Salinity 22	48	
					Salinity 28	120	
					**Hypoosmotic treatment**		
					Salinity 8 (acclimatization to artificial water)	24	
					Salinity 6	48	
					Salinity 2.5	48	
					Salinity 0.5	120	
Hiddensee, Vitter Bodden	S6	54°34′40″N, 13°06′48″E	120	Mesohaline Salinity 10	**Hyperosmotic treatment**		0
					Salinity 8 (acclimatization to artificial water)	24	
					Salinity 16	48	
					Salinity 22	48	
					Salinity 28	120	
					**Hypoosmotic treatment**		
					Salinity 8 (acclimatization to artificial water)	24	
					Salinity 6	48	
					Salinity 2.5	48	
					Salinity 0.5	120	

Collected snails were kept in 54 L aquariums filled with water sampled at the collection site for at least 24 h at room temperature. These storage aquariums were aerated and illuminated by daylight lamps (Hagen Sun Glo fluorescent tube T8; 15 W) operating in a 24 h light:dark cycle of 15:9 h. Natural food was provided by adding biofilm-covered pebbles collected at the collection sites of the snails to the aquariums.

The salinity manipulation experiments were conducted in small glass aquariums in which nine snails per aquarium were held during the transfer experiments with 1 L of water and pebbles that snails fed on for 10 days at room temperature (Table 1).

**FIGURE 1:**
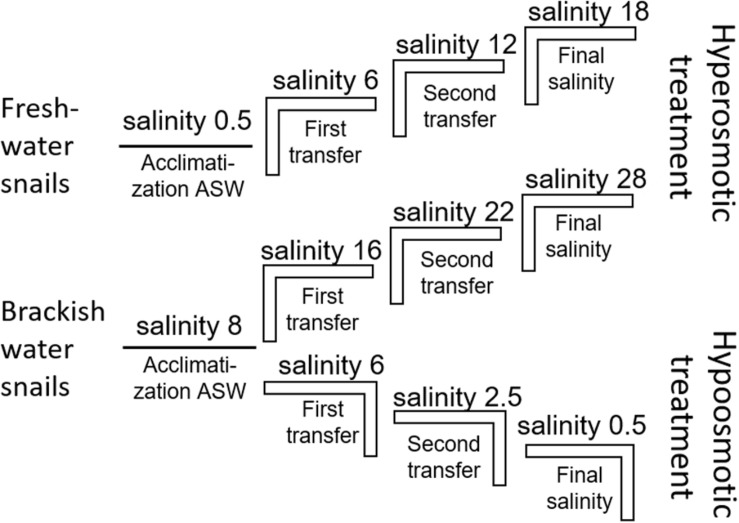
Experimental setup scheme for the stepwise hyperosmotic and hypoosmotic manipulations (ASW = artificial seawater).

The artificial seawater (ASW) in the manipulation experiments was prepared using Tropic Marin aquarium salt (Hünenburg, Switzerland) to 10 L distilled water (Supplementary Table S1). The experimental setup consisted of control aquaria containing artificial water where the ambient salinity was adjusted (ASW control). In addition to the ASW control aquaria also *in situ* control (IS control) samples were prepared. For the hypo- and hyperosmotic manipulations, ASW was prepared in a stepwise manner as described in Wiesenthal et al. (2018) (Figure 1). For hyperosmotic treatment with snails sampled from freshwater, the salinity was raised from 0.5 to 18 and with snails sampled from brackish water the salinity was raised from 8 to 28 (Tables 1, 2 and Figure 1). For hypoosmotic manipulation with snails from brackish water, the salinity was decreased from 8 to 0.5. For each transfer step (Figure 1), pebbles and snails were transferred to a new clean aquarium. Water exchange took place during each transfer step of the manipulations. The salinity was constantly checked with a Vapro 5520 osmometer (Wescor Inc., Logan, UT, United States) and conductivity meter (VWR International, LLC, United States). During the experiment, the snails were checked daily for activity. They were considered to be alive, if they stuck to surfaces or movement of the foot muscle was observed. After a total of 10 days, the snails were collected from final salinities (*n* = 71 + *in situ* control *n* = 30, in total *n* = 101).

The snails were cooled to 4°C for 15 min before dissecting the gut. The shell parts were removed with forceps, and the mantel was opened to remove the gastrointestinal tract under a stereomicroscope (Supplementary Figure S2). Samples contained the whole digestive tract which consists of buccal mass, pharynx, esophagus, intestine, anus, stomach, and midgut gland. While dissecting the gastrointestinal tract, symbiotic *Oligochaetes* were recorded and the snail biological sex was identified (46 females, 55 males). The gastrointestinal tract was transferred into a 2 mL reaction tube containing 100–300 μL RNAlater (Qiagen). The samples were incubated for >6 h at 4°C and frozen at −20°C until use.

### DNA Extraction and Sequence Processing

DNA was extracted according to the modified protocols from Weinbauer et al. (2002) and Lueders et al. (2004). Dichlorodimethylsilane treated glass beads (0.5 g diameter 0.5 mm and 3 diameter 3 mm) were added to 2 mL tubes together with the digestive tract of one snail. 750 μL 120 mM NaPO_4_ buffer (pH 8) and 250 μL TNS [500 mM Tris-HCl pH 8.0, 100 mM NaCl, 10% sodium dodecyl sulfate (SDS) (wt/vol)] were added and bead beat for 3 min at 2,000 r/min (Mikro-dismembrator U, B. Braun Biotech International). Following this, the samples were incubated at 65°C for 1 h followed by another round of bead beating for 3 min at 2,000 r/min. After centrifuging at 14,000 r/min for 5 min, the supernatant was transferred to a new 2 mL tube. A mixture of phenol:chloroform:isoamyl alcohol (25:24:1) at pH 8 was then added to the supernatant and carefully mixed. After centrifugation at 14,000 r/min for 5 min, the upper aqueous phase of the supernatant was placed in a new 2 mL tube and 1 vol of chloroform:isoamyl (24:1) was added and carefully mixed. After further centrifugation at 14,000 r/min for 12 min, the upper aqueous phase of the supernatant was transferred to a new 1.5 mL tube and 2 μL RNase (100 mg/mL) (Qiagen) was added and incubated at 37°C for 30 min. Ice-cold isopropanol was added to the sample and inverted a couple of times and incubated at room temperature for 15 min. After centrifugation at 14,000 r/min for 20 min, the supernatant was removed and 250 μL of 95% ethanol was added. After centrifugation at 14,000 r/min for 5 min, the ethanol was removed and the remaining pellet was dried at 50°C until the ethanol evaporated (∼5–15 min). The pellet was resuspended in 50 μL AE (10 mM Tris-Cl 0.5 mM EDTA; pH 9.0) buffer (Qiagen). Both the amount and quality of the DNA was controlled with a NanoDrop^TM^ UV–Vis spectrophotometer. For bacterial community analysis, the DNA was PCR amplified according to the protocol of Herlemann et al. (2011), using 30 cycles to amplify bacterial sequences using the primers Bakt_341F and Bakt_805R. The amplicons were purified using PCR Kleen (Bio-Rad), tags added according to the protocol provided by the sequencing company and sent to FIMM at the University of Helsinki, Finland. A total of 7,353,519 reads were generated by Illumina sequencing using PE250 chemistry.

The resulting sequences were quality checked using Trimmomatic (V0.36) to remove Illumina-specific sequences and regions with low-sequence quality (average quality score < Q20). PCR primers were removed using the default values in Cutadapt (V2.3). The reads were paired (16 bp overlap, minimum length 300 bp) and quality trimmed using the VSEARCH tool (Rognes et al., 2016). These were then taxonomically assigned using the SILVA next-generation sequencing (NGS) pipeline (Glöckner et al., 2017) based on SILVA release version 132 (Pruesse et al., 2007). SILVA NGS performs additional quality checks according to SINA-based alignments (Pruesse et al., 2012) with a curated seed database in which PCR artifacts or non-SSU reads are excluded. The longest read serves as a reference for taxonomic classification using a BLAST (version 2.2.30+) search against the SILVA SSURef dataset. The classification of the reference sequence of each cluster (98% sequence identity) is then mapped to all members of the respective cluster and to their replicates. SILVA NGS was able to classify a total of 3,881,648 high quality reads (2% were rejected by the quality control) for a 66 samples. Non/bacterial sequences such as chloroplasts, mitochondria, eukaryotes, and *Archaea* were excluded because the primer set employed in the analysis only has a very limited coverage of these groups resulting in 3,658,589 sequences of which 153,566 were taxonomically not assigned (“no relative”).

The raw reads were deposited at the NCBI SRA under bioproject number PRJNA587055.

For internal transcribed spacer 2 (ITS2) analysis of *T. fluviatilis*, the DNA were PCR amplified according to a protocol from Vinarski et al. (2011) using the primers LT1 (Bargues et al., 2001) and ITS2-Rixo (Almeyda-Artigas et al., 2000). The temperature profile used was 94°C 4 min (94°C 30 s, 56°C 30 s, 72°C 1 min) × 35, 72°C 7 min, 8°C. The amplicon was purified using PCR Kleen (Bio-Rad) and Sanger sequenced by the sequencing facility at the Tartu University, Estonia.

The sequences from the ITS2 region were quality checked using the software Chromas and, together with an ITS2 reference sequence obtained from a *T. fluviatilis* EST library. Low quality sequences were discarded. All sequences were imported into ARB (Ludwig et al., 2004) to calculate a maximum likelihood phylogenetic tree (PhyML). The topology of the tree was tested separately by neighbor-joining and parsimony analysis (DNAPARS) using a bootstrapping algorithm (seqboot; 100 bootstraps).

The reads were deposited at EBI under accession number LR736795-LR736837.

### Statistical Analysis

The number of reads per sample varied between 6517 and 169,699 reads and, because of this large variation, was normalized by cumulative sum scaling (CSS) using the R package metagenomeSeq (Paulson et al., 2013). Richness was estimated using the R package phyloseq. A Kruskal–Wallis test and a *post hoc* Tukey’s pairwise test were used to calculate significant differences between the numbers of OTUs in the samples. Variations in bacterial community structure were characterized in a principal coordinates analysis (PCoA) using the Bray–Curtis dissimilarity in the “vegan” community ecology package of R (Oksanen et al., 2013) and PAST software package version 3.22 (Hammer et al., 2001). Bacterial communities were correlated according to environmental parameters using the *envfit* package included in the R package “vegan.” A linear discriminant analysis effect size (LEfSe) analysis (Segata et al., 2011) was used to identify bacterial groups whose relative abundance differed significantly between samples. For this purpose, the default setting with the multi-class analysis the “One against all” was used. OTUs identified in the LEfSe as significantly enriched were defined as indicator OTUs.

Prediction of functional profiles and functional redundancy of prokaryotic communities from 16S rRNA gene sequences was estimated using the R package Tax4Fun2 (Wemheuer et al., 2018). Functional profiles were initially aligned against the supplied 16S rRNA reference sequences by BLAST using the runRefBlast function (database SILVA Ref99NR) and functional predictions were subsequently calculated using its makeFunctionalPrediction function resulting in function annotation according to the KEGG database for prokaryotes (July 2018 release), which served as a reference database.

## Results

### Host Internal Transcribed Spacer 2 Analysis

**FIGURE 2:**
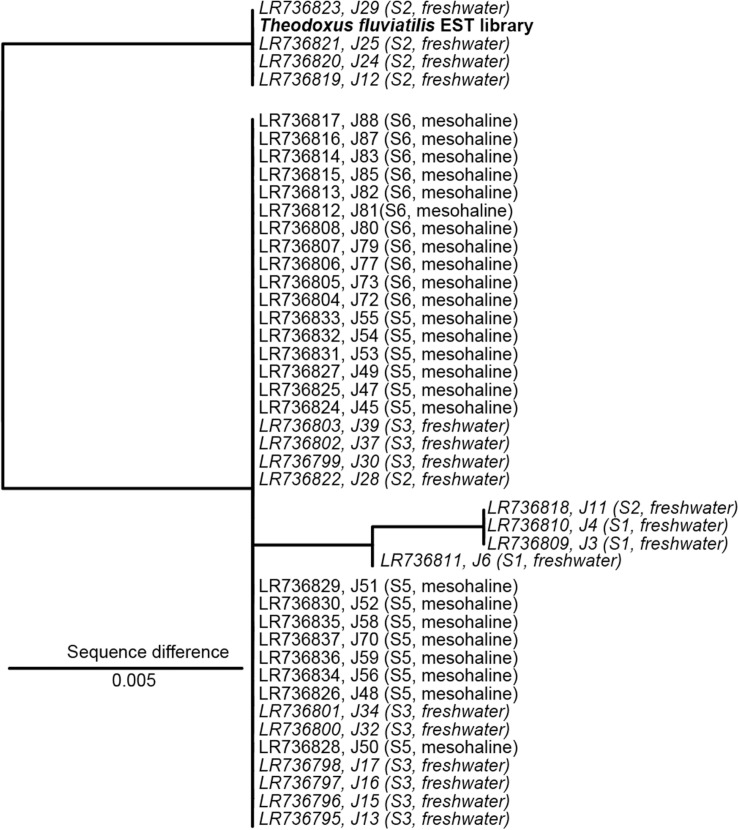
Nuclear marker internal transcribed spacer 2 (ITS2) sequence maximum likelihood tree based on 398 sequence columns without outgroup. A sequence from *Theodoxus fluviatilis* transcriptome analysis (EST library) was used as reference (marked in bold). Freshwater samples are indicated in italics. The scale bar indicates a 0.5% sequence difference. J29, J25, J24, J12 with 1% sequence difference J6, J11, J4, J3 with 0.5% sequence difference.

The analysis of the ITS2 biomarker regions confirmed that all samples belong to *T. fluviatilis* (Figure 2). The majority of the ITS2 sequences were identical, samples J11, J4, J3, and J6 showed only minor and random differences to the main group (>99.5% sequence similarity). However, a distinct group of sequences covering samples from Lake Schmaler Luzin, south end (J29, J25, J24, J12) contained characteristic sequence motives that differed from the other sequences (99% sequence similarity, Figure 2 and Supplementary Figure S3). The slight differences in some ITS2 sequences from *T. fluviatilis* samples from Lake Schmaler Luzin (south end) caused no significant effect in the gastrointestinal microbiome (Supplementary Figure S4) and the samples were therefore included in the analysis as the other *T. fluviatilis* samples.

### Microbiome Analysis

**FIGURE 3:**
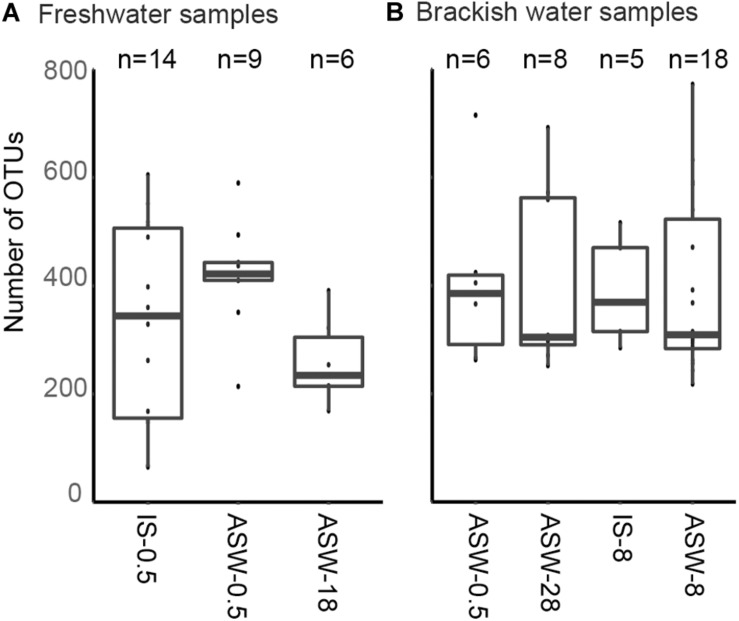
Observed number of operational taxonomic units (OTUs) *in situ* and during manipulation. Freshwater samples **(A)**: freshwater *in situ* salinity (IS-0.5), artificial seawater control (ASW-0.5) where salinity was adjusted to freshwater salinity, artificial seawater raised to 18 for freshwater snails hyperosmotic treatment (ASW-18). Brackish water samples **(B)**: brackish water *in situ* salinity (IS-8), artificial seawater control where salinity was adjusted to salinity 8 (ASW-8), artificial seawater raised to salinity 28 for brackish water snails hyperosmotic treatment (ASW-28), artificial seawater with salinity 0.5 for brackish water snails hypoosmotic treatment (ASW-0.5). The box indicates the 25–75% quartiles; the median is given by the horizontal line inside the box. The minimal and maximal values are shown with short vertical lines and samples are shown as dots.

SILVA NGS classified the sequences in a total of 1,964 operational taxonomic units (OTUs) on genus level among 42 different phyla. The number of OTUs detected in the freshwater snail microbiome at ambient conditions was 65–605 OTUs and in the mesohaline snail microbiome between 218 and 772 OTUs (Figure 3A). Transferring freshwater snails to salinity 18 resulted in an insignificant (*p* = 0.882) decrease in the number of OTUs (169–391 OTUs). Transferring mesohaline water snails from salinity 8 to freshwater or to salinity 28 also did not significantly influence the number of OTUs (266–715 OTUs, 252–692 OTUs accordingly; Figure 3B).

### Changes in the Bacterial Community Composition

**FIGURE 4:**
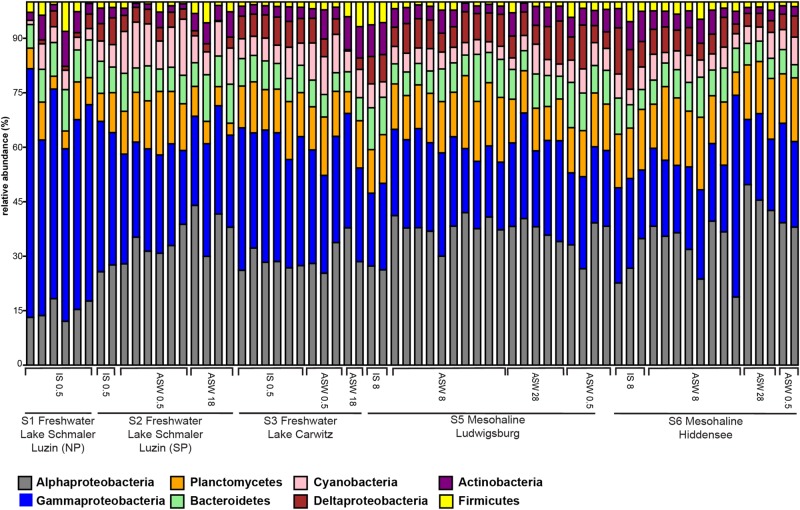
Bar graph of most abundant OTU-s on Phylum/Class level. Freshwater sampling sites: freshwater *in situ* salinity (IS-0.5), artificial seawater control (ASW-0.5) where salinity was adjusted to freshwater salinity, artificial seawater raised to 18 for freshwater snails hyperosmotic treatment (ASW-18). Brackish water samples: brackish water *in situ* salinity (IS-8), artificial seawater control where salinity was adjusted to salinity 8 (ASW-8), artificial seawater raised to salinity 28 for brackish water snails hyperosmotic treatment (ASW-28), artificial seawater with salinity 0.5 for brackish water snails hypoosmotic treatment (ASW-0.5).

**FIGURE 5:**
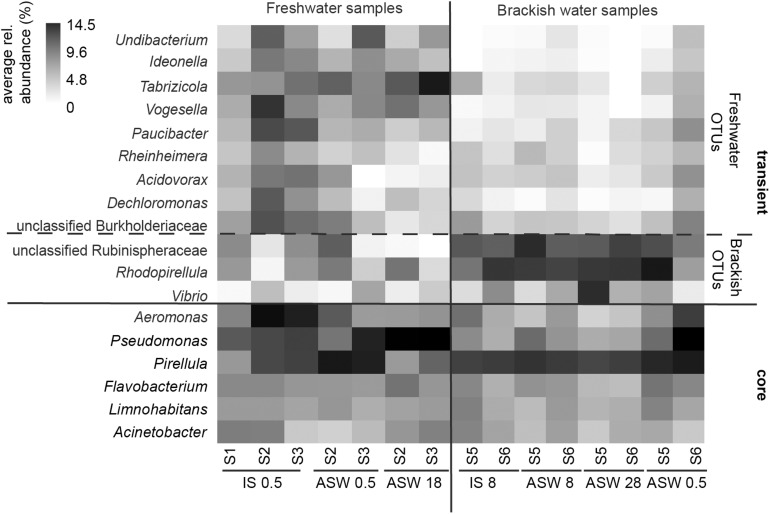
Heatmap of core microbiome and significantly different OTU-s for mesohaline and freshwater samples according to Lefse results. Freshwater samples: freshwater *in situ* salinity (IS-0.5), artificial seawater control (ASW-0.5) where salinity was adjusted to freshwater salinity, artificial seawater raised to 18 for freshwater snails hyperosmotic treatment (ASW-18). Brackish water samples: brackish water *in situ* salinity (IS-8), artificial seawater control where salinity was adjusted to salinity 8 (ASW-8), artificial seawater raised to salinity 28 for brackish water snails hyperosmotic treatment (ASW-28), artificial seawater with salinity 0.5 for brackish water snails hypoosmotic treatment (ASW-0.5).

On phylum/class level, the differences between freshwater, mesohaline, and manipulated snail microbiomes were minor (Figure 4). Gammaproteobacteria (relative abundance 15.4–63.3%) and Alphaproteobacteria (relative abundance 11.1–46.2%) were the most dominant bacterial classes in all samples, however, Bacteroidia (relative abundance 3.2–11.5%), Oxyphotobacteria (relative abundance 1.1–13.3%), and Actinobacteria (relative abundance 0.4–8.2%) as well as Planctomycetacia (relative abundance 2.1–16.6%) were also found in high abundance. The relative abundance of the dominant OTUs in freshwater snail samples were, on average, *Aeromonas* (0.8%), *Pseudomonas* (0.8%), *Pirellula* (0.7%), *Tabrizicola* (0.6%), and unclassified Rhizobiales (0.6%). Snails biological sex did not influence the bacterial community composition (*F* = 1.01, *p* = 0.375). When freshwater snails were transferred to salinity 18, the bacterial community composition was still dominated by *Pseudomonas* (1.2%), *Aeromonas* (0.9%) and unclassified Rhizobiaceae (0.8%), but Hoeflea (1.0%) and Erythrobacter (1.0%) became more abundant in the community composition. The dominant genera in brackish water snails samples were *Rhodopirellula* (0.7%), *Pirellula* (0.7%), *Blastopirellula* (0.7%), unclassified Rubinisphaeraceae (0.7%), and *Cyanobium* (0.6%). In contrast with freshwater snail microbiomes, hyperosmotic and hypoosmotic manipulation of the mesohaline snail microbiomes did not result in significantly different OTUs (Figure 5). The transfer to salinity 28 resulted in a bacterial community resembling the ambient brackish snails bacterial community with *Rhodopirellula* (1.0%), *Pirellula* (1.0%), *Blastopirellula* (1.0%) still dominating the community, only *Vibrio* (1.0%) and unclassified Rubinisphaeraceae (0.9%) became abundant. Similar to this result, the hypoosmotic treatment (salinity 0.5) resulted in no cardinal changes in the bacterial community composition. The dominant genera were still *Pirellula* (0.8%), *Rhodopirellula* (0.8%), *Blastopirellula* (0.7%), and unclassified Rubinisphaeraceae (0.7%). *Pseudomonas* (0.7%) became notably abundant as well. Over all a core microbiome consisting of *Flavobacterium* (1.6%), *Pseudomonas* (0.8%), *Pirellula* (0.7%), *Limnohabitans* (0.5%), and *Acinetobacter* (0.4%) were present in all *T. fluviatilis* samples independent of the saline manipulation and habitat.

**FIGURE 6:**
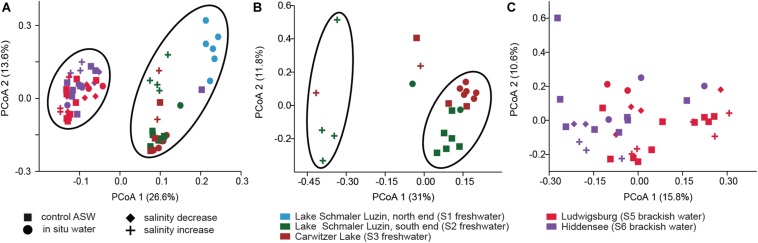
Principal Coordinate Analysis based on Bray–Curtis dissimilarity of bacterial community composition on OTU level. **(A)** All samples, **(B)** freshwater samples from S2 (dark green) and S3 (brown) at ambient conditions (filled dot) and control conditions (filled square) and after saline manipulation (plus). **(C)** Brackish samples from S5 (pink) and S6 (purple) at ambient conditions (filled dot) and control conditions (filled square) and after saline manipulation (plus—salinity increase and filled diamond—salinity decrease).

Despite the continuous presence of the most abundant bacterial genera (core microbiome), we identified typical OTUs using LEfSe. According to LefSe analysis, the freshwater samples included *Undibacterium*, *Ideonella*, *Tabrizicola*, *Vogesella*, *Paucibacter*, *Rheinheimera*, *Acidovorax*, *Dechloromonas*, and unclassified Burkholderiaceae whereas unclassified Rubinisphaeraceae, *Rhodopirellula*, *Vibrio*, and *Aeromonas* were more typical for the mesohaline samples (Figure 5). To visualize the differences in composition between the bacterial communities in freshwater and mesohaline snails under both ambient conditions and during manipulation, we employed principal coordinate analysis (PCoA) (Figure 6A). The PCoA indicates that ambient brackish water snail microbiome samples and ambient freshwater snail microbiomes are separated on the first PCo (26.6% difference explained) and a subsequent ANOSIM test validated the significance of this difference between these habitats (*p* < 0.01; *R* = 0.38). When increasing the salinity for freshwater snails to salinity 18, we observed a clear shift in the gut microbiome (Figure 6B). Changing the salinity of the mesohaline snails to either freshwater or salinity 28 did not result in a significant shift in the bacterial community (Figure 6C).

### Identification of Potential Functional Clusters

**FIGURE 7:**
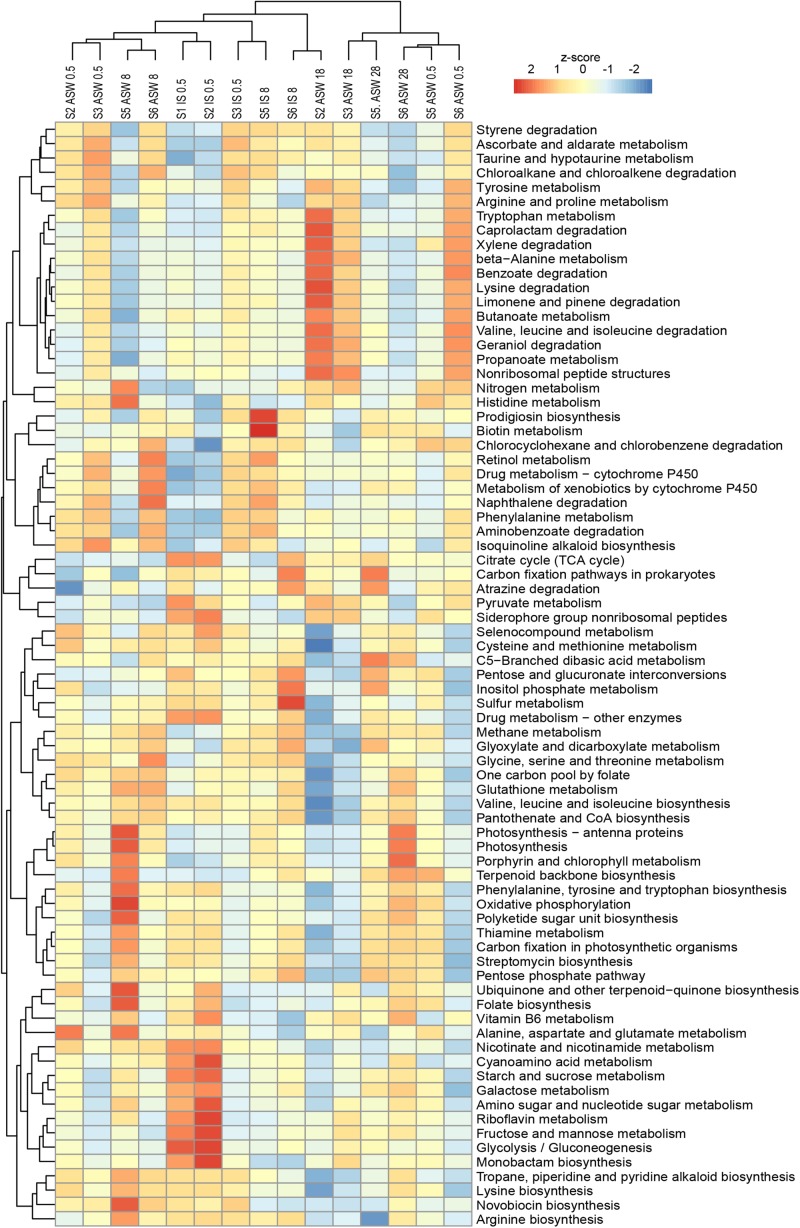
Variations of KEGG metabolic pathways in the functional microbial communities (with the threshold 5%). The heatmap shows the functional profiles based on the relative abundance of metabolic pathways after a z-score transformation. Freshwater samples: freshwater *in situ* salinity (IS-0.5), artificial seawater control (ASW-0.5) where salinity was adjusted to freshwater salinity, artificial seawater raised to 18 for freshwater snails hyperosmotic treatment (ASW-18). Brackish water samples: brackish water *in situ* salinity (IS-8), artificial seawater control where salinity was adjusted to salinity 8 (ASW-8), artificial seawater raised to salinity 28 for brackish water snails hyperosmotic treatment (ASW-28), artificial seawater with salinity 0.5 for brackish water snails hypoosmotic treatment (ASW-0.5). Samples are clustered based on similarity.

In the functional analysis, we concentrated on the level 3 category “Metabolism” and included the subcategories “Biosynthesis of other secondary metabolites,” “Xenobiotics biodegradation and metabolism,” “Metabolism of cofactors and vitamins,” “Carbohydrate metabolism,” “Amino acid metabolism,” and “Energy metabolism.” Functional predictions of these categories using Tax4fun2 suggest significant differences between the freshwater and brackish water digestive tract microbiomes (*F* = 5.579, *p* < 0.01). The function prediction showed that the ascendant functions for overall gastrointestinal microbiota were related to “Amino acid metabolism” whereas “Leucine and isoleucine degradation,” “Valine metabolism,” and “Glycine, serine, and threonine metabolism” were more pronounced. The category “Energy metabolism” was dominated by the subcategories “Oxidative phosphorylation” and “Sulfur metabolism” (Figure 7). A one-way PERMANOVA test showed significantly different functions between sample features (IS control, ASW control, salinity manipulation) and bacterial community functions (*F* = 6.874, *p* < 0.01).

With regard to the bacterial community composition (Figure 3), the freshwater samples in their ambient conditions from Lake Schmaler Luzin (S1, S2) were similar in their functions and ascendant functions for gastrointestinal microbiota were related to the category “Energy metabolism” and “Carbohydrate metabolism” including “Pyruvate metabolism” and “Glyoxylate and dicarboxylate metabolism.” After the salinity manipulation, the Lake Schmaler Luzin south end (S2) gastrointestinal microbiota was enriched compared to control samples with functions that were related to “Pyruvate metabolism” including “Glyoxylate and dicarboxylate metabolism” but also to “Butanoate metabolism,” “Propanoate metabolism,” and “Amino sugar and nucleotide sugar metabolism.” The functions of IS control samples did not differ significantly compared to the salinity manipulated group (*p* = 0.064); however, functional changes were significant between the IS control and ASW control (*F* = 3.59, *p* = 0.035) and between the ASW control and salinity manipulation group (*F* = 14.52, *p* < 0.01). Freshwater samples from Lake Carwitz (S3) gastrointestinal microbiota under control conditions (IS control and ASW control) and after the manipulation had ascendant functions related to “Valine, leucine, and isoleucine degradation” and “Glyoxylate and dicarboxylate metabolism.” There were no significant changes between the IS control and ASW control group (*p* = 0.154) or between the IS control and salinity manipulation group (*p* = 0.109). Also, there was no significant difference between the ASW control and salinity manipulated groups (*p* = 0.597).

Ludwigsburg (S5) brackish water snails gastrointestinal microbiota ascendant functions for IS control and ASW control were related to “Glyoxylate and dicarboxylate metabolism,” “Pyruvate metabolism,” and “Oxidative phosphorylation.” After manipulation to salinity 28, the gastrointestinal microbiota functions remained almost the same; however, “Stilbenoid, diarylheptanoid and gingerol biosynthesis” became more pronounced compared to control samples. Changes between the ASW control and the salinity manipulation group were salinity was up to salinity 28 showed statistically significant changes (*F* = 7.818, *p* < 0.01). Hypoosmotic manipulation to salinity 0.5 resulted in insignificant functional changes compared to the IS control (*p* = 0.595), ASW control (*p* = 0.052), and increased salinity group (*p* = 0.475). There were no significant changes between the IS control and ASW control groups (*p* = 0.062) as well.

Vitter Bodden (S6) brackish water snails gastrointestinal microbiota ascendant functions for IS control and ASW control were related to “Glyoxylate and dicarboxylate metabolism” and “Glycine, serine, and threonine metabolism.” After the hyperosmotic manipulation (salinity 28), the functions related to “Stilbenoid, diarylheptanoid, and gingerol biosynthesis” and “Isoflavonoid biosynthesis” became more pronounced compared to control groups. After hypoosmotic manipulation (salinity 0.5), the gastrointestinal microbiota functions were again related to “Glyoxylate and dicarboxylate metabolism” and “Glycine, serine, and threonine metabolism.” Changes between the ASW control and the hypoosmotic manipulation group (salinity 0.5) showed statistically significant changes (*F* = 7.563, *p* = 0.025); however, there were no statistically significant changes between the other analyzed groups.

LefSe analysis of significantly different functions between samples indicated that the hyperosmotic manipulation of the samples correlated with the pathways for “Arginine and proline metabolism” and “Amino sugar and nucleotide sugar metabolism.”

## Discussion

Salinization of freshwater environments is expected due to a global rate of mean sea-level rise with an average rate of rise since 1993 of +3.2 mm (±0.4 mm) year^–1^ (Nicholls and Cazenave, 2010). In combination with reduced rainfall, sea level rise causes saltwater intrusion in current freshwater environments altering low lying freshwater environments to brackish environments (Neubauer et al., 2012). Significant changes in salinity are likely to result in the loss/change of key microorganism (Herlemann et al., 2011), changes in microbial metabolism (Neubauer, 2013), and nutrient cycling (Marton et al., 2012). The effect of changing salinity on the digestive tract microbiome in host systems is currently unknown. The aim of this study was to investigate the responses of a host-protected bacterial community to shifts in salinity using *T. fluviatilis* as a model system. *T. fluviatilis* snails were obtained from different sites with either mesohaline water (salinity 8) or freshwater conditions. Analysis of the highly variable ITS2 sequence from the host revealed that all snails belong to the species *T. fluviatilis* (Figure 2). Therefore, potential effect due to differences in host phylogeny can be excluded. This was expected because the North German populations of *T. fluviatilis* have been described as a single population (Bunje, 2005). However, few sequences obtained from Lake Schmaler Luzin (south end) suggest the presence of a subpopulation. These potential phylogenetic differences had no effect on the bacterial community composition and both phylogenetic lineages were found in close proximity at Lake Schmaler Luzin (south end).

The number of OTUs (alpha-diversity) was comparable in both ambient and salinity manipulated samples that originated from both freshwater and mesohaline habitats (Figure 3). Only an insignificant reduction was observed for the transfer of snails from ASW control to salinity 18 (Tukey test, *p* > 0.05). Changes in salinity have been observed to cause a reduction in macroinvertebrate richness (Remane, 1934) and phytoplankton diversity (Olli et al., 2019). Yet, the bacterial richness in both pelagic and benthic salinity gradients is rather constant (Herlemann et al., 2011, 2016; Klier et al., 2018). In a whole ecosystem manipulation experiment, Berga et al. (2017) also concluded that alpha-diversity measure of bacterial communities were resistant to changes in salinity. Previous research in host associated systems showed that the freshwater–saltwater change caused an insignificant decrease in the number of microbiome bacteria in *Salmo salar* L. (Dehler et al., 2017). Therefore, similar to pelagic (Herlemann et al., 2016) and benthic environments (Klier et al., 2018), changes in the bacterial species richness in host-protected environments seem to be connected with other parameters than salinity.

The bacterial community profiling revealed a diverse community based on 16S rRNA gene sequences, which were predominantly derived from Gammaproteobacteria, Alphaproteobacteria, Planctomycetes, Bacteroidetes, and Actinobacteria (Figure 4). The high abundance of Gammaproteobacteria and Alphaproteobacteria has been previously found in invertebrate gut microbiomes including *Achatina fulica* (Pawar et al., 2012), Diplopoda, *Cylindroiulus fulviceps* (Knapp et al., 2009), and oysters (King et al., 2012). Despite the dominance of Proteobacteria in all samples, the results on finer taxonomic levels (OTUs) indicate that the composition of the bacterial gut community in freshwater and mesohaline *T. fluviatilis* is significantly different (Figure 5). This confirms our first hypothesis suggesting that *T. fluviatilis* obtained at different salinities have significantly different bacterial community compositions. It also supports previous studies showing that salinity is a major environmental factor which causes changes in the bacterial community composition and impairs ecosystem functions (Herlemann et al., 2011; Weston et al., 2011; Neubauer, 2013).

Although hosts can be colonized by opportunistic food-related or widespread environmental taxa, they are often directly or indirectly colonized by microbiota released in the environment by conspecifics (Engel and Moran, 2013). LefSe analysis indicated that *Undibacterium*, *Ideonella*, *Tabrizicola*, *Vogesella*, *Paucibacter*, *Rheinheimera*, *Acidovorax*, *Dechloromonas*, and unclassified Burkholderiaceae were more pronounced in freshwater samples and unclassified Rubinisphaeraceae, *Rhodopirellula*, and *Vibrio* were more pronounced within mesohaline samples (Figure 5). Several of these OTUs have the ability to support the digestion of algal food for *T. fluviatilis* suggesting functional redundancy of these freshwater and mesohaline specific OTUs. The genus *Paucibacter* contains many species with the ability to degrade microcystins and nodularin (Pheng et al., 2017) a toxin typically found in the presence of cyanobacteria. *Undibacterium* are cellulolytic bacteria (Eder et al., 2011) and *Vogesella* sp. has genes for protocatechuate degradation (Woo et al., 2017), a substance often synthesized in higher plants. *Rhodopirellula*, which was mainly found in mesohaline samples, has been shown to break down sulfated polysaccharides (Glöckner et al., 2003) that are often part of algae and diatoms. *Vibrios* are among the most commonly reported groups of gut bacteria in marine vertebrates and invertebrates (Harris, 1993; Sawabe, 2006) and are able to degrade cellulose. This suggests that several OTUs in freshwater and mesohaline *T. fluviatilis* digestive systems are involved in enzymatic food digestion.

In addition to these specific OTUs, we also found OTUs that are highly abundant in freshwater and mesohaline gut microbiomes, independent of the salinity manipulation. The most conspicuous were *Aeromonas* and *Pseudomonas* that are often highly abundant in freshwater and mesohaline gut microbiomes (Yasuda and Kitao, 1980; Dempsey et al., 1989; Liu et al., 2011). The wide distribution of *Aeromonas* in different aquatic environments such as freshwater, estuarine, and seawater (Kaper et al., 1981), sewage, and wastewater (Boussaid et al., 1991), underlines the capacity of this species to adapt to environments that differ in terms of nutrients or the presence of other aquatic microorganisms thereby suggesting a very general lifestyle. *Aeromonas* and *Pseudomonas* have previously been identified as a cellulolytic species (Sindhu and Dadarwal, 2001; Jiang et al., 2011) and were also abundantly found in all samples. Other OTUs constantly found were *Pirellula*, *Flavobacterium*, *Limnohabitans*, and *Acinetobacter*, all of which have a large variety of enzymes with degradation capacities. *Acinetobacter* and *Pseudomonas* are commonly detected in gut systems of other aquatic animals such as fish and crabs (Huber et al., 2004). As cellulolytic bacteria, *Acinetobacter* has the ability to produce xylanase (Ekperigin, 2007). *Pirellula* spp. were also found to dominate in the guts of diatom-fed abalone (Nel et al., 2017). Although betaproteobacteria, like *Limnohabitans*, are rather irrelevant in the digestive tracts of most animals, they appear to be typical intestinal bacteria in *Daphnia* and may even maintain a symbiotic relationship with their host (Freese and Schink, 2011). A CARD–FISH analysis of gut homogenates of environmental *Daphnia pulex* also revealed Betaproteobacteria as one major group (Peter and Sommaruga, 2008). *Limnohabitans* were also a dominant bacterial group associated with a Cladocera (*Bosmina*) (Grossart et al., 2009). In our study, the most abundantly found genus from the phylum Bacteroidetes was *Flavobacterium.* Members of the genus *Flavobacterium* are widely distributed in nature, occurring mostly in aquatic ecosystems ranging in salinity from freshwater to seawater. It is also one of the most commonly reported genera of gut bacteria in aquatic invertebrates (Harris, 1993). Several flavobacterial freshwater species are potentially the etiological agents of fish diseases. Other *Flavobacterium* species appear to be harmless, chemoheterotrophic species that play a role in mineralizing various types of organic matter (carbohydrates, amino acids, proteins, and polysaccharides) in aquatic ecosystems. Some species in the family Flavobacteriaceae degrade soluble cellulose derivatives such as carboxymethylcellulose or hydroxyethylcellulose but not all *Flavobacterium* species are cellulolytic (Dworkin et al., 2006).

These five bacterial genera (*Pseudomonas, Pirellula*, *Flavobacterium*, *Limnohabitans*, and *Acinetobacter*) in the gut microbiome of *T. fluviatilis* may constitute a core microbiome (Shade and Handelsman, 2012) consisting of generalists able to cross salinity barriers (Székely and Langenheder, 2014). The presence of a core microbiome supports our third hypothesis; however, many of the OTUs seem to be transient and strongly influenced by changes in salinity. Therefore, the gut microbiota in *T. fluviatilis* has a multilayered structure, composed of both a core microbiota that is likely under host control and a flexible pool of microbes modulated by the environment. The host controlled core microbiome is likely to have an important function in the host (Johnston and Rolff, 2015).

Our second hypothesis is that an artificial increase in salinity results in a respective shift in the microbiome. When freshwater snails were exposed to increased salinity (salinity 18), the bacterial composition changed significantly (*F* = 3.066, *p* < 0.01) (Figure 6B). The generalists (*Aeromonas* and *Pseudomonas*) were still abundantly found, which is consistent to Székely and Langenheder (2014) findings that habitat generalists are more likely to be assembled by dispersal-related mechanisms because they can tolerate a wide range of environmental conditions. In addition to that, two OTUs (*Hoeflea* and *Erythrobacter*) became more abundant. Neither of these were found in the mesohaline snails; however, *Hoeflea* which is from family *Rhizobiaceae* and *Erythrobacter* which is from family *Sphingomonadaceae* are widespread bacteria found in the water column and biofilm (Ishii et al., 2008; Huang et al., 2013).

Ambient mesohaline *T. fluviatilis* gut samples were dominated by *Pirellula*, *Rhodopirellula*, *and Blastopirellula* from the phylum Planctomycetes (Figure 5). *Rhodopirellula* and *Blastopirellula* are more adapted to saltwater habitats, but *Pirellula* is found abundantly in both marine (Gebers et al., 1985; Kölbel-Boelke et al., 1985; Schlesner, 1986) and freshwater habitats (Staley, 1973; Tekniepe et al., 1981; Schlesner, 1994). When mesohaline snails were put through hyperosmotic manipulation (salinity 28), most of the genera found in the control group (*Rhodopirellula*, *Pirellula*, *Blastopirellula*, and unclassified Rubinisphaeraceae) were still present, only *Cyanobium* became less abundant, and *Vibrio* became more abundant. *Vibrio* is described as an alginolytic and salt-tolerant species (Sawabe et al., 1998; Kisand et al., 2005). Similar to hyperosmotic manipulation, the microbiome during hypoosmotic manipulation (salinity 0.5) changed only slightly. The abundant *Pseudomonas*, *Pirellula*, *Rhodopirellula*, *Blastopirellula*, *Aeromonas*, and unclassified *Rubinisphaeraceae* were still the main genera.

The minor response of the mesohaline gastrointestinal microbiome to changes in salinity (Figure 6C) indicates that the mesohaline microbiome is more resistant to shifts in salinity. This also suggests the presence of generalist bacteria that thrive over a wide range of salinities. In contrast, the response of the freshwater microbiome to an increase in salinity was significant indicating that these bacteria are more specialized to freshwater conditions. An increase in salinity for the freshwater snails caused a different bacterial community than found in the mesohaline snails and resulted in a shift in the associated functions (see below). This could indicate that the host may prevent polyhaline adapted bacteria from establishing in the digestive system microbiome to avoid a loss in essential functions. Another explanation could be that the *T. fluviatilis* digestive microbiome is best adapted to intermediate salinities since it originates from the mesohaline Black Sea. Our results indicate that in the host-protected environment, changes in salinity have a smaller influence on the bacterial community than previously shown for unprotected, pelagic bacteria (e.g., Shen et al., 2018). The extent of the saline protection seems to depend on the environmental history of the host.

The functional predictions using 16S rRNA genes have significant limitations because they can only identify certain taxa by the chosen set of primers and rely on described functions in the databases. However, using 16S rRNA as a predictor for microbial functions has the advantage to avoid host contamination obscure the microbial signal. Despite several limitations, hypotheses about changes in functional properties can be made (Langille, 2018). The changes we observed in the microbial community composition during hyperosmotic manipulation of freshwater samples did not result in major changes in predicted functions. In contrast, the minor changes in microbial community of mesohaline samples resulted in significant functional changes. Langille (2018) described that functions seem to be more conserved across samples than across taxa, which suggest that functions are more resilient across communities than the individual strains that can be lost or gained.

The salinity manipulation of all samples correlated with the pathways for “Alanine, arginine, and proline metabolism,” “Aspartate and glutamate metabolism,” and “Amino sugar and nucleotide sugar metabolism” suggesting a specific response of osmolyte production from the bacterial community. Already in 1961, Allen showed that free alanine, glutamic acid, and aspartic acid concentrations in a brackish water clam increased with increasing ambient salinity. Euryhaline mollusks mainly accumulate amino acids as organic osmolytes in their cells under hyperosmotic stress (Shumway et al., 1977; Pierce and Amende, 1981). *T. fluviatilis* from both freshwater and mesohaline conditions showed the ability to accumulate organic osmolytes in response to hyperosmotic stress equally well; however, they differ in the pathways of acquiring these organic osmolytes (Wiesenthal et al., 2019). The main constituents of the increased amounts of organic osmolytes are alanine and proline that seem to be most important for an initial coping with high environmental salinity conditions and were also among the most important amino acid pathways that responded in the microbiome. Xu et al. (2017) described the noticeably more pronounced amino sugar and nucleotide sugar metabolism functions when the euryhaline decapod *Litopenaeus vannamei* was put through a salinity stress experiment. Taken together, the digestive tract microbiome of snails may also support the amino acid supply for the hosts osmolyte production during salinity stress.

While dissecting the guts, we found Oligochaeta in the snail shells. The Oligochaeta specimens were only detected within freshwater samples as also described by Fashuyi and Williams (1977). The salinity manipulation where freshwater snails were exposed to a salinity 18 resulted in a complete loss of Oligochaeta. None of the freshwater snails from hyperosmotic aquariums had any of the specimens present after the salinity manipulation experiment. The Oligochaeta found belong to the genus *Chaetogaster limnaei limnaei. C. limnaei* is an ectosymbiont and is present inside the mantle cavity of the snail, whereas the parasitic form *C. limnaei vaghini* lives in the kidney of the snail (Smythe et al., 2015). It has been confirmed that *C. limnaei limnaei* has a mutualistic relationship with freshwater snails such as *Galba truncatula* (Muñiz-Pareja and Iturbe-Espinoza, 2018). The absence of the *Oligochaeta* after salinity treatment is explained by the worms strict adaptation to freshwater, it simply did not survive the rise in salinity (Muñiz-Pareja and Iturbe-Espinoza, 2018).

Polysaccharides are the most important energy source for the algal feeders, and our results support the hypothesis that the digestive tract microbiome supplies the host with polysaccharide degrading enzymes. At hyperosmotic and hypoosmotic manipulation, mesohaline *T. fluviatilis* seemed to influence the gut microenvironment to maintain the original bacterial community with high cellulolytic potential and the ability to produce osmolytes. This indicates that the host can compensate for the strong effects of salinity on the gut microbiome. However, a hyperosmotic manipulation of *T. fluviatilis* induced by transferring animals from freshwater to salinity 18 resulted in a shift in the bacterial community composition that was not compensated by the host, suggesting that freshwater snails are more sensitive to changes in salinity. Therefore, salinization of coastal freshwater environments due to sea level rise can influence the gut microbiome of this snail with currently unknown consequences for the host. More studies on host associated systems in the freshwater-saline transition will be necessary to validate this concept.

## Data Availability Statement

The datasets generated for this study can be found in the NCBI BioProject under the accession numbers PRJNA587055 and LR736795-LR736837.

## Author Contributions

JK sampled snails, dissected the gastrointestinal tract, performed the experiments, and generated the image of the gastrointestinal tract. CK did DNA extraction. CK and VK did preparation for sequencing. CK, VK, and DH did statistical and bioinformatic analysis. DH did ITS sequencing and phylogenetic analysis. HT submitted sequences. HT and KK contributed to the lab work. J-PH and DH planned the experiments. All authors contributed to the writing of the manuscript.

## Conflict of Interest

The authors declare that the research was conducted in the absence of any commercial or financial relationships that could be construed as a potential conflict of interest.
