# Supplementary material for: Impact of Salinity on the Gastrointestinal Bacterial Community of Theodoxus fluviatilis
Source: Front Microbiol. 2020 May 8;11:683. doi: 10.3389/fmicb.2020.00683 (PMC7225522; doi:10.3389/fmicb.2020.00683)
Supplement: Supplementary file 1 [file Data_Sheet_1.pdf]

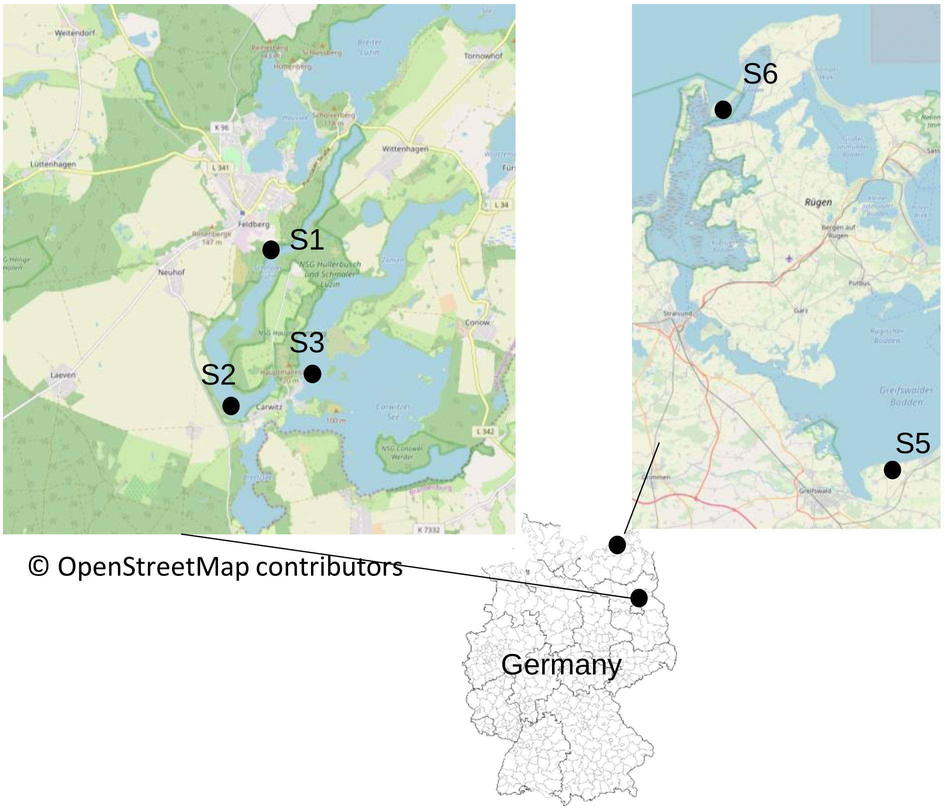

S1: Lake Schmaler Luzin, north end
S2: Lake Schmaler Luzin, south end
S3: Carwitzer Lake
S5: Ludwigsburg, Greifswalder Bodden
S6: Hiddensee, Vitter Bodden

Supplementary Figure 1. Sampling locations of *Theodoxus fluviatilis* in Germany.

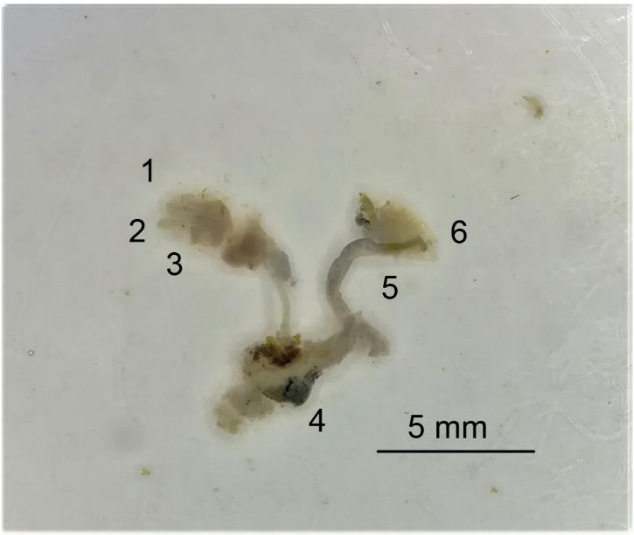

Supplementary Figure 2. Morpho-anatomy of the digestive tract of Theodoxus fluviatilis and the different gastrointestinal compartments: anterior tract (1 - buccal mass, 2 - pharynx, 3 - oesophagus, 4 - stomach) and posterior tract (5 - intestine, 6 - anus).

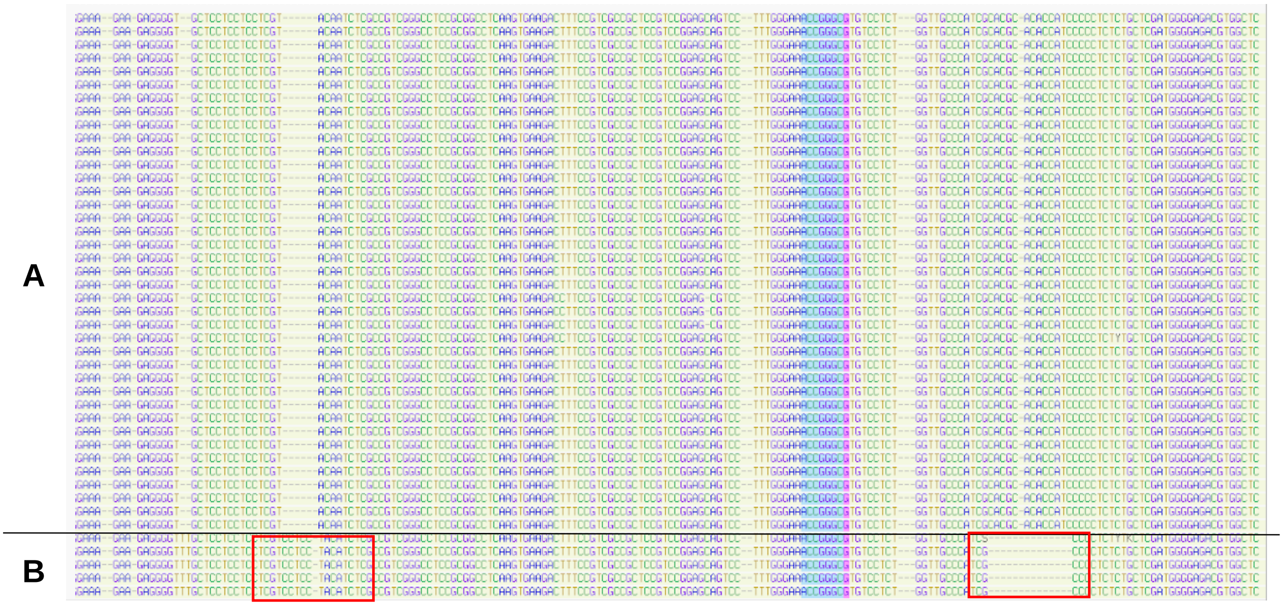

Supplementary Figure 3. Sequence motives (red frame) of *Theodoxus fluviatilis* ITS2 sequences. (A) main phylogenetic lineage (B) four sequences from Lake Schmaler Luzin (south).

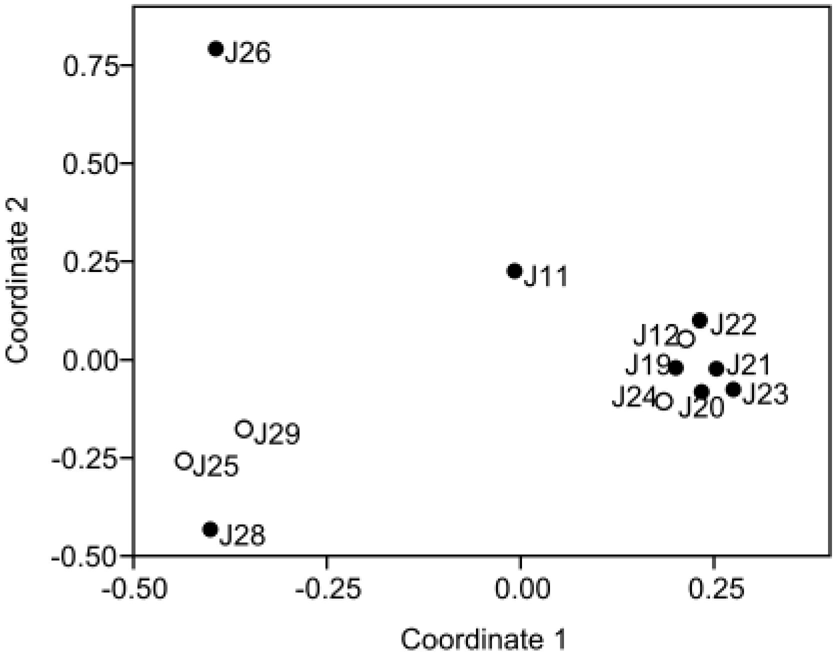

Supplementary Figure 4. Principle Coordinate Analysis (PCoA) of *Theodoxus fluviatilis* microbiome samples from Lake Schmaler Luzin. Samples from the potential subpopulation are empty dots other are filled dots (see Figure 2).

Supplementary Table 1. Preparation of artificial seawater (ASW).

Artificial water salinity for transfer steps	Amount of aquarium salt in g per 10 L of artificial water
Salinity 0.5	5
Salinity 2.5	25
Salinity 6	60
Salinity 8	80
Salinity 12	120
Salinity 16	160
Salinity 18	180
Salinity 22	220
Salinity 28	280
